# The Effect of Therapeutic Lumbar Punctures on Acute Mortality From Cryptococcal Meningitis

**DOI:** 10.1093/cid/ciu596

**Published:** 2014-07-23

**Authors:** Melissa A. Rolfes, Kathy Huppler Hullsiek, Joshua Rhein, Henry W. Nabeta, Kabanda Taseera, Charlotte Schutz, Abdu Musubire, Radha Rajasingham, Darlisha A. Williams, Friedrich Thienemann, Conrad Muzoora, Graeme Meintjes, David B. Meya, David R. Boulware

**Affiliations:** 1 Department of Medicine, Medical School; 2 Division of Biostatistics, School of Public Health, University of Minnesota, Minneapolis; 3 Infectious Diseases Institute, Makerere University, Kampala; 4 Internal Medicine, Faculty of Medicine, Mbarara University of Science and Technology, Uganda; 5 Institute of Infectious Disease and Molecular Medicine and Department of Medicine, University of Cape Town, South Africa; 6 Department of Medicine, Imperial College London, United Kingdom;; 7 School of Medicine, College of Health Sciences, Makerere University, Kampala, Uganda

**Keywords:** HIV, cryptococcal meningitis, epidemiology, mortality, therapeutic lumbar punctures

## Abstract

***Introduction.*** Cryptococcal meningitis is the most common cause of adult meningitis in sub-Saharan Africa. Raised intracranial pressure (ICP) is common in cryptococcosis. Prior studies suggest elevated ICP is associated with mortality, and guidelines recommend frequent lumbar punctures (LPs) to control ICP. However, the magnitude of the impact of LPs on cryptococcal-related mortality is unknown.

***Methods.*** In sum, 248 individuals with human immunodeficiency virus (HIV)-associated cryptococcal meningitis, screened for the Cryptococcal Optimal ART Timing (COAT) trial in Uganda and South Africa, were observed. Individuals received an LP to diagnose meningitis, and subsequent therapeutic LPs were recommended for elevated ICP (>250 mmH_2_O) or new symptoms. We compared survival, through 11 days, between individuals receiving at least 1 therapeutic LP with individuals not receiving therapeutic LPs. The COAT trial randomized subjects at 7–11 days; thus, follow-up stopped at time of death, randomization, or 11 days.

***Results.*** Seventy-five (30%) individuals had at least 1 therapeutic LP. Individuals receiving therapeutic LPs had higher cerebrospinal fluid (CSF) opening pressures, higher CSF fungal burdens, and were more likely to have altered mental status at baseline than those with no therapeutic LPs. Thirty-one deaths (18%) occurred among 173 individuals without a therapeutic LP and 5 deaths (7%) among 75 with at least 1 therapeutic LP. The adjusted relative risk of mortality was 0.31 (95% confidence interval: .12–.82). The association was observed regardless of opening pressure at baseline.

***Conclusions.*** Therapeutic LPs were associated with a 69% relative improvement in survival, regardless of initial intracranial pressure. The role of therapeutic LPs should be reevaluated.

## Abstract (precis)

Intracranial pressure management with repeat lumbar puncture (LP) was investigated in patients with cryptococcal meningitis in sub-Saharan Africa. Conducting at least 1 additional LP soon after cryptococcal diagnosis was related to decreased risk of acute mortality regardless of initial pressure.

**(See the Editorial Commentary by Pappas on pages 1615–17.)**

Despite recognition of the burden and advancements in treatment, acute mortality from human immunodeficiency virus (HIV)-associated cryptococcal meningitis remains high with 17%–50% mortality within 2 weeks of diagnosis among individuals in sub-Saharan Africa [1–9]. One complication of cryptococcal meningitis is elevated intracranial pressure (ICP), defined as a cerebral spinal fluid (CSF) opening pressure >250 mmH_2_O, and prior literature suggests there is higher mortality among cryptococcal patients with raised ICP [10–12].

Raised ICP is common at the time of diagnosis and frequently leads to changes in mental status, headache, loss of vision and hearing, or death. Aggressive management of ICP is therefore suggested in treatment guidelines for cryptococcal meningitis, including daily therapeutic lumbar punctures (LPs) until pressures and symptoms have normalized [13, 14]. With these recommendations, elevated ICP typically resolves over the first 2 weeks of antifungal therapy. Prior studies have not found an association between baseline opening pressure and 2-week mortality attributed, in part, to aggressive control of ICP with therapeutic LPs [4, 15, 16]. A recent comparison to historical data also suggested that following a strict schedule of therapeutic LPs may have led to lower 30-day mortality in a hospital in Tanzania [17]. We aimed to add to the current body of literature and estimate the direct effect of therapeutic LPs on acute mortality in a prospective cohort of HIV-infected individuals with cryptococcal meningitis in Uganda and South Africa.

## METHODS

### Study Population

Data from the Cryptococcal Optimal ART Timing (COAT) trial, conducted from November 2010 to April 2012, and an observational cohort of patients with cryptococcal meningitis, from April 2012 through December 2012, were used in this analysis. Ethical approval was granted from the Uganda National Council of Science and Technology, South African Medicines Control Council, and the Institutional Review Boards at the University of Minnesota, Makerere University, University of Cape Town, and Mbarara University of Science and Technology.

The COAT trial was a randomized clinical strategy trial of early antiretroviral therapy (ART) initiation (1 week after cryptococcal meningitis diagnosis) compared to deferred ART initiation (5 weeks after cryptococcal meningitis diagnosis; www.clinicaltrials.gov: NCT01075152). Individuals with suspected meningitis were recruited from 3 sites: Mulago National Referral Hospital in Kampala, Uganda; Mbarara National Referral Hospital in Mbarara, Uganda; and GF Jooste Hospital in Cape Town, South Africa. Individuals participating in COAT were randomized within 7–11 days of cryptococcal treatment initiation. Observational cohort recruitment occurred at Mulago Hospital after enrollment in the COAT trial ended. Individuals in the observational cohort received identical care to COAT participants but with deferred ART initiation.

HIV-infected, ART-naive individuals were eligible for enrollment into the trial or cohort if they were at least 18 years old, provided written informed consent, had cryptococcal meningitis documented by CSF culture or cryptococcal antigen test, and were receiving amphotericin-based treatment. Individuals on antifungal therapy for >1 week or with a prior episode of cryptococcal meningitis were excluded. Antifungal induction treatment included 2 weeks of amphotericin B deoxycholate (0.7–1.0 mg/kg/day) plus fluconazole (800 mg/day). Baseline clinical and laboratory features were collected at the time of cryptococcal meningitis diagnosis.

For this analysis, follow-up time began the day after diagnosis of cryptococcal meningitis in order to allow individuals the opportunity for a therapeutic LP. For individuals screened for the COAT trial, observation ended at the time of death or at randomization (7–11 days after starting treatment for cryptococcal meningitis) because the COAT protocol indicated a scheduled LP be performed at randomization and at 14 days. The median time of COAT randomization was 8 days from the start of antifungal therapy. For individuals in the observational cohort, observation ended at the time of death or after 11 days of follow-up.

### Lumbar Punctures and CSF Parameters

Therapeutic LPs after the diagnostic LP were recommended for those with baseline CSF opening pressure >250 mmH_2_O or symptoms of raised pressure [14]. Participants could receive multiple therapeutic LPs at the discretion of the attending clinician; however, in this analysis, exposure was defined as receiving at least 1 therapeutic LP. Written informed consent was provided for the initial diagnostic LP, and verbal consent was required before all subsequent therapeutic LPs. Participants or their surrogate had the right to refuse therapeutic LPs. The amount of CSF removed during an LP was recorded, and CSF opening and closing pressures were measured whenever the study team performed the LP.

### Statistical Analysis

Because exposure status was not known at the start of observation, receipt of therapeutic LPs was included as a time-varying exposure in analysis. Individuals contributed person-time to the “no therapeutic LP” group after their diagnostic LP and until they received a therapeutic LP, died, or were censored (at COAT trial randomization or a maximum of 11 days of follow-up). Once a therapeutic LP was performed, individuals contributed person-time to the “therapeutic LP” group. Crude mortality rates were calculated for person-time before a therapeutic LP, as well as for person-time after the first therapeutic LP. Differences in baseline factors by eventual LP and vital status were compared using χ^2^ and Wilcoxon rank-sum tests.

The outcome of interest was all-cause mortality within 11 days of follow-up. Time to death was modeled with a pooled Poisson regression model. Inverse probability weights were used to control for confounding (see Supplementary Appendix). The relative risk of mortality was estimated, comparing the “therapeutic LP” group with the “no therapeutic LP” group [18, 19].

Possible confounders were considered among baseline characteristics associated with exposure, known to be related to acute mortality, and that changed the estimated relative risk by >10% after adjustment. Linear, nonlinear, and categorical forms of continuous variables were considered. Linear terms were chosen for all variables except Glasgow Coma Scale (GCS) scores, which were dichotomized as <15 (indicating altered mental status) or 15 for all models.

*P* values <.05 were considered statistically significant. All data analyses were conducted in SAS version 9.3 (SAS Institute, Cary, NC).

### Missing Data

Multiple imputation was conducted to account for missing baseline characteristics (see Supplementary Appendix). The most common missing characteristic was weight (34% missing), due to the inability of critically ill individuals to stand for measurement. Other common missing factors were CSF opening pressure (16% missing) and serum potassium (12% missing). All other variables were missing in less than 10% of the data. Reasons for missing data stemmed from insufficient supplies or specimen volumes and were thus assumed to be missing at random [20]; with the exception of weight, which could be related to severity of illness and short-term mortality. Sensitivity analyses considered models with an indicator variable for missing weight but were found not to change the overall conclusions.

## RESULTS

### Study Population

**Figure 1.**
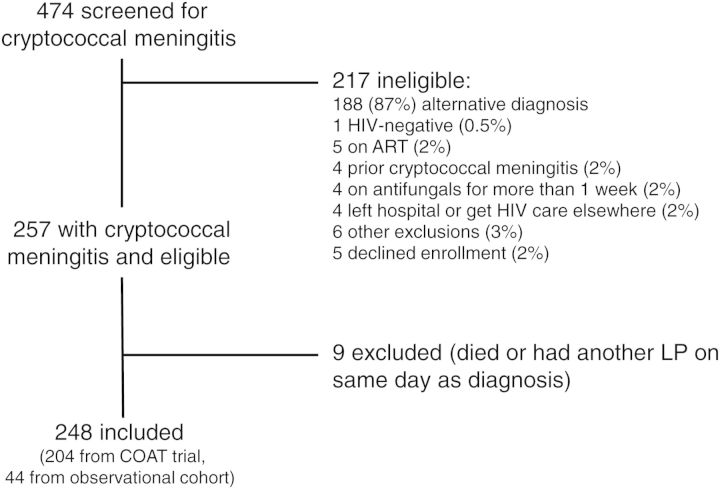
Selection of cohort participants among HIV-infected individuals in South Africa and Uganda screened for cryptococcal meningitis. Abbreviations: ART, antiretroviral therapy; COAT, Cryptococcal Optimal ART Timing; HIV, human immunodeficiency virus; LP, lumbar puncture.

Four hundred seventy-four individuals were screened, and 257 were found to have cryptococcal meningitis and were considered for inclusion in this analysis. Nine individuals died or had a therapeutic LP on the same day as diagnosis and were excluded from further analysis, leaving 248 individuals for analysis (Figure 1). Included individuals were all HIV-infected and observed for a total of 1698 person-days (median follow-up 7 days [interquartile range [IQR]: 6–8 days]). The median age of the cohort was 36 years, median duration of headache was 2 weeks before diagnosis, 55% were male, and 29% had altered mental status (GCS <15).

**Figure 2.**
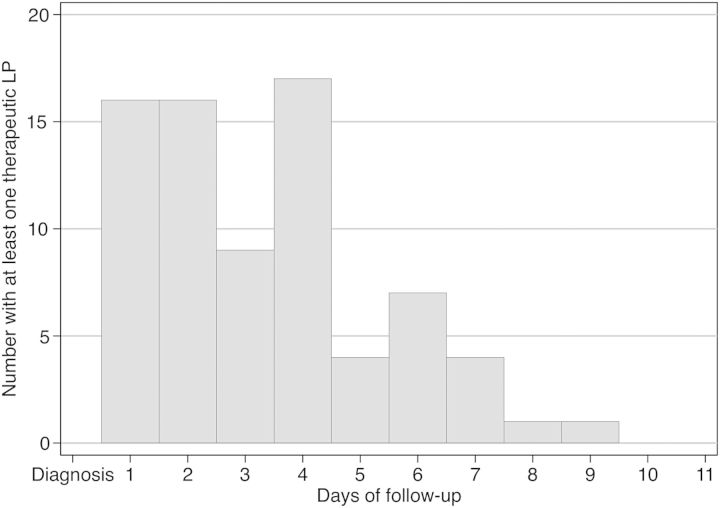
Time of the first therapeutic LP in days from diagnosis of cryptococcal meningitis, among 75 HIV-infected individuals in South Africa and Uganda who received at least 1 LP after an initial diagnostic LP. The median time after diagnosis until the first therapeutic LP was 3 days. Abbreviations: HIV, human immunodeficiency virus; LP, lumbar puncture.

Seventy-five (30%) subjects received at least 1 therapeutic LP after diagnosis. The median baseline ICP at diagnosis was 269 mmH_2_O (IQR: 180–373 mmH_2_O) and was significantly higher among individuals who later received a therapeutic LP (Table 1). The majority of individuals (80%) receiving a therapeutic LP underwent only 1 LP during follow-up, although 15 individuals had ≥2 LPs including 2 individuals who received 7 LPs and 1 individual who received 8 LPs during the observation period. The median time to first therapeutic LP was 3 days after diagnosis (IQR: 2–4 days; Figure 2).

**Table 1.** Baseline Characteristics and Mortality by Therapeutic Lumbar Punctures Among HIV-Infected Individuals With Cryptococcal Meningitis in South Africa and Uganda

	At Least 1 Therapeutic LP		No Therapeutic LP		
	N With Data	Median [IQR] or N (%)^a^	N With Data	Median [IQR] or N (%)^a^	*P* Value^b^
N per group		75 (30)		173 (70)	
Follow-up time (days)		7 [6, 9]		7 [6, 8]	
Site^c^	75		173		.02
Kampala		61 (34)		120 (66)	
Mbarara		4 (11)		34 (89)	
Cape Town		10 (35)		19 (65)	
Age (years)	75	34 [29, 40]	173	37 [30, 42]	.15
Males	75	44 (59)	173	91 (53)	.38
Weight (kg)	43	57 [46, 62]	121	52 [45, 57]	.08
Missing weight	75	32 (43)	173	52 (30)	.05
Headache duration	72		169		.56
<7 d		8 (11)		18 (11)	
7–13 d		27 (38)		50 (30)	
14–20 d		16 (22)		35 (21)	
≥21 d		21 (29)		66 (39)	
Papilledema	71	0 (0)	163	8 (5)	.06
Karnofsky score	74	50 [40, 50]	173	50 [40, 60]	.16
Glasgow Coma Scale <15	74	26 (35)	173	45 (26)	.15
Heart rate (per minute)	74	76 [66, 90]	172	81 [72, 97]	.01
Respiratory rate (per minute)	71	20 [20, 24]	172	22 [20, 24]	.40
Temperature >37.5°C, axillary	74	9 (12)	171	40 (23)	.04
Clinical laboratory values					
Hemoglobin (g/dL)	71	11.5 [9.4, 13.0]	155	11.0 [8.9, 13.0]	.41
Creatinine (mg/dL)	73	0.6 [0.5, 0.8]	160	0.7 [0.6, 0.9]	.03
CSF parameters					
Opening pressure (mmH_2_O)	69	346 [220, 440]	139	248 [150, 338]	<.001
Opening pressure >250 mmH_2_O	69	48 (70)	139	69 (50)	.007
Closing pressure (mmH_2_O)	64	100 [80, 137]	126	90 [60, 120]	.04
Amount of CSF removed (mL)	72	19 [12, 27]	168	14 [8, 20]	<.001
Quantitative cryptococcal CSF culture (log_10_ CFU/mL)	75	5.3 [4.4, 5.6]	159	5.0 [3.9, 5.5]	.03
White blood cells (/μL of CSF)^d^	40	93 [36, 310]	92	67 [25, 135]	.08
White blood cells <5 cells/μL	74	34 (46)	158	66 (42)	.55
Outcome					
Died	75	5 (7)	173	31 (18)	
Mortality rate (per 100 person-days)^e^		1.3 (95% CI, .4–3.0)		2.4 (95% CI, 1.6–3.3)	.19

Abbreviations: CFU, colony-forming unit; CI, confidence interval; CSF, cerebrospinal fluid; HIV, human immunodeficiency virus; IQR, interquartile range; LP, lumbar puncture.

^a^ Median and IQR. Frequency and percentages are column percentages.

^b^ *P* values from χ^2^ test for frequencies and Wilcoxon rank-sum test for medians.

^c^ Row percentages are presented.

^d^ Among those with detectable CSF white blood cell count (≥5 cells/μL).

^e^ All individuals contributed person-time to the no therapeutic LP group until he/she received a therapeutic LP, at which point the individual contributed person-time to the therapeutic LP group. Total follow-up time was 395 person-days after individuals received their first therapeutic LP and 1314 person-days prior to the first therapeutic LP. Confidence intervals are exact CIs and *P* value from a crude, unweighted Poisson regression model.

At the first therapeutic LP, the median opening pressure was 270 mmH_2_O (IQR: 180–401 mmH_2_O) overall, and higher than 250 mmH_2_O in 58% of persons. At the first therapeutic LP, ICP was higher among those with initially elevated ICP at diagnosis; median opening pressure was 329 mmH_2_O (IQR: 210–430 mmH_2_O) among those with opening pressure >250 mmH_2_O at diagnosis, and 255 mmH_2_O (IQR: 160–375 mmH_2_O) among those with opening pressure <250 mmH_2_O at diagnosis.

The occurrence of therapeutic LPs was slightly different by study site, with LPs occurring more frequently in Kampala and Cape Town. Those receiving additional LPs had higher CSF fungal burden, higher CSF opening and closing pressures, and more CSF volume was removed during the first diagnostic LP. Other clinical and demographic characteristics were generally similar between the groups.

### Acute Mortality

Thirty-six deaths occurred during observation for an overall mortality rate of 2.1 per 100 person-days (95% confidence interval [CI], 1.5–2.9 per 100 person-days). The median time to death was 4 days (IQR: 2–6 days). Acute mortality was associated with lower weight, missing weight at baseline, lower GCS, greater heart rate, faster respiratory rate, and higher CSF fungal burden at cryptococcal meningitis diagnosis (Table 2). The CSF opening pressure, amount of CSF removed, and CSF white blood cell counts at the diagnostic LP were similar between those who died and those surviving through follow-up.

**Table 2.** Baseline Characteristics by Vital Status Among HIV-Infected Individuals With Cryptococcal Meningitis in South Africa and Uganda

	Died		Survived Observation		
	N With Data	Median [IQR] or N (%)^a^	N With Data	Median [IQR] or N (%)^a^	*P* Value^b^
N per group		36 (15)		212 (85)	
Follow-up time (days)		4 [2, 6]		7 [6,8]	
Site^c^	36		212		.22
Kampala		31 (17)		150 (82)	
Mbarara		3 (8)		35 (92)	
Cape Town		2 (7)		27 (93)	
Age (years)	36	38 [30, 44]	212	35 [29, 40]	.10
Males, N (%)	36	20 (56)	212	115 (54)	.88
Weight (kg)	15	43.2 [40.0, 54.0]	149	54.0 [46.0, 59.7]	.007
Missing weight	36	21 (58)	212	63 (30)	.001
Karnofsky score	36	50 [40, 50]	211	50 [40, 60]	.02
Glasgow Coma Scale <15	36	16 (44)	211	55 (26)	.03
Heart rate (per minute)	36	92 [76, 106]	210	80 [70, 90]	.003
Respiratory rate (per minute)	35	24 [20, 26]	208	20 [20, 24]	.03
CSF parameters					
Opening pressure (mmH_2_O)	27	290 [150, 392]	181	265 [180, 370]	.92
Opening pressure >250 mmH_2_O	27	16 (59)	181	101 (56)	.74
Closing pressure (mmH_2_O)	25	78 (54, 110)	165	100 (70, 130)	.08
Amount of CSF removed (mL)	34	18 (10,23)	206	15 (10, 23)	.47
Quantitative cryptococcal CSF culture (log_10_ CFU/mL)	32	5.3 [4.6, 5.8]	202	5.1 [4.0, 5.5]	.04
White blood cells (/μL of CSF)^d^	15	45 [25, 135]	117	79 [30, 180]	.35
White blood cells <5 cells/μL	33	18 (55)	199	82 (41)	.15

Abbreviations: CFU, colony-forming unit; CSF, cerebrospinal fluid; HIV, human immunodeficiency virus; IQR, interquartile range.

^a^ Median and IQR. Frequency and percentages are column percentages.

^b^ *P* values from χ^2^ test for frequencies and Wilcoxon rank-sum test for medians.

^c^ Row percentages are presented.

^d^ Among those with detectable CSF white blood cell count (≥5 cells/μL).

**Figure 3.**
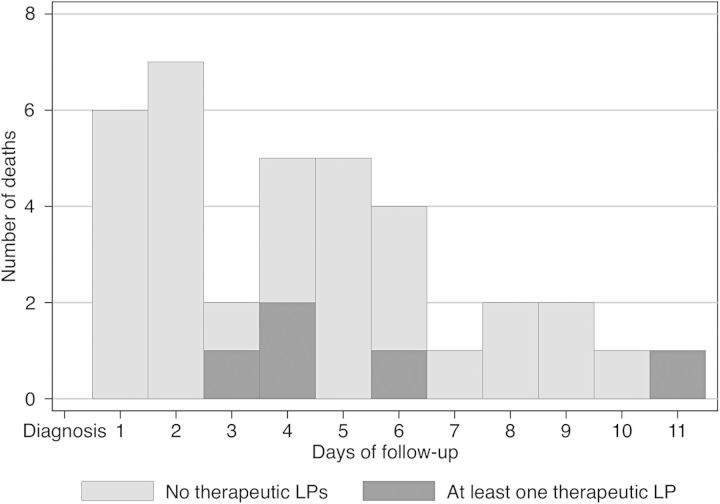
Distribution of time to death among those with and without at least 1 therapeutic LP among HIV-infected individuals with cryptococcal meningitis in South Africa and Uganda. A total of 36 deaths occurred within 11 days after diagnosis with cryptococcal meningitis. The overall median time to death was 4 days after diagnosis. Abbreviations: HIV, human immunodeficiency virus; LP, lumbar puncture.

Deaths in the group who received a therapeutic LP occurred later during observation than the deaths among those who did not receive additional LPs (Figure 3). Prior to receiving a therapeutic LP, the mortality rate was 2.4 per 100 person-days (95% CI, 1.6–3.3 per 100 person-days) compared to 1.3 per 100 person-days (95% CI, .4–3.0 per 100 person-days) after receiving a therapeutic LP. Of those who received a therapeutic LP and died, all 5 individuals underwent only 1 additional LP during observation.

### Multivariable Association

The crude, unweighted relative risk (RR) of mortality was 0.5 (95% CI, .2, 1.4), comparing those with at least 1 compared to those with no therapeutic LP (Table 3). Adjustment for heart rate, CSF fungal burden, and an indicator for low GCS into the weighted model resulted in more extreme relative risks (adjusted models 1–3). After adjusting for heart rate, CSF fungal burden, and GCS (adjusted model 3), the average effect of therapeutic LPs was to reduce the risk of mortality by 69% (95% CI, 18%–88%). Additional adjustment for CSF opening pressure did not result in measureable changes in the relative risk (0.3; 95% CI, .1, 1.0). Adjustment for baseline CSF closing pressure and weight did not alter the estimated effect.

**Table 3.** Estimated Relative Risk of Acute Mortality After Receiving a Therapeutic Lumbar Puncture in HIV-Infected Individuals With Cryptococcal Meningitis in South Africa and Uganda

	Relative Risk	95% CI	Mean sw_*i*_ (SD)^a^
Crude, unweighted model	0.53	(.20, 1.37)	…
Marginal Structural Model Pooled Poisson Regression^b^			
Adjusted model 1	0.50	(.19, 1.32)	1.01 (0.22)
Adjusted model 2	0.39	(.14, 1.07)	1.01 (0.29)
Adjusted model 3	0.31	(.12, .82)	1.02 (0.52)

Abbreviations: CI, confidence interval; GCS, Glasgow Coma Score; sw*_i_*, stabilized weight; HIV, human immunodeficiency virus; SD, standard deviation.

^a^ Stabilized weights (sw*_i_*) are the product of the stabilized exposure and stabilized censoring weights.

^b^ Adjusted model 1 is adjusted for heart rate. Adjusted model 2 is adjusted model 1 additionally adjusted for cerebrospinal fluid fungal burden. Adjusted model 3 is adjusted model 2 additionally adjusted with an indicator for GCS <15.

### Opening Pressure Subgroups

Exploratory analysis was conducted to assess whether the effect of therapeutic LPs differed by baseline CSF opening pressure. The frequency of therapeutic LPs was higher in the subgroup with high baseline pressures than in those with lower pressures (Table 4); however, there was little evidence of heterogeneity of the relative risk estimates, suggesting that the effect of therapeutic LPs on acute mortality did not differ by baseline opening pressure.

**Table 4.** Association of Therapeutic Lumbar Puncture and Acute Mortality in HIV-Infected Individuals With Cryptococcal Meningitis by Baseline CSF Opening Pressure

	At Least 1 Therapeutic LP	No Therapeutic LP	Overall
Baseline CSF opening pressure <250 mmH_2_O			
Number of individuals (% of overall)	21 (23%)	70 (77%)	91
Deaths, N (%)	0 (0%)	11 (16%)	11 (12%)
Person-days of observation	98	554	652
Mortality rate (per 100 person-days)	0	1.99	1.69
Unadjusted relative risk (95% CI)	0.00 [.00, 2.25]^a^		
Baseline CSF opening pressure ≥250 mmH_2_O			
Number of individuals (% of overall)	48 (41%)	69 (59%)	117
Deaths, N (%)	4 (8%)	12 (17%)	16 (14%)
Person-days of observation	260	505	765
Mortality rate (per 100 person-days)	1.54	2.38	2.09
Unadjusted relative risk (95% CI)	0.65 [.15, 2.14]		
Baseline CSF opening pressure not available			
Number of individuals (% of overall)	6 (15%)	34 (85%)	40
Deaths, N (%)	1 (17%)	8 (24%)	9 (23%)
Person-days of observation	28	253	281
Mortality rate (per 100 person-days)	3.57	3.16	3.20
Unadjusted relative risk (95% CI)	1.13 [.03, 8.42]		

Abbreviations: CI, confidence interval; CSF, cerebrospinal fluid; HIV, human immunodeficiency virus; LP, lumbar puncture.

^a^ A value of 0.5 was added to both the numerator and denominator for the group with at least 1 therapeutic LP in order to estimate the rate and confidence intervals.

Forty individuals did not have CSF opening pressure measured at the time of diagnosis, primarily because non-study staff conducted the initial LP without the use of a manometer. Baseline characteristics were similar among those who did and did not have opening pressure measured (results not shown), except that a greater amount of CSF was removed during the diagnostic LP in those with measured opening pressure (median 8 mL removed in those without manometer measurement [IQR: 5–15 mL] vs 16 mL in those with measurements [IQR: 10–25 mL], *P* < .001). Of those with missing opening pressure data, 15% of individuals received therapeutic LPs during follow-up. Overall, mortality was highest among those without pressure measurements (23%) compared to individuals with measured pressures (13%). The sample size was too small to draw definitive conclusions on the effect of therapeutic LPs on mortality in those without measured CSF opening pressure.

## DISCUSSION

In this study, 30% of patients with HIV-associated cryptococcal meningitis received at least 1 therapeutic LP; overall, 15% died within 11 days of follow-up. The majority of those who died (85%) did not have a therapeutic LP during observation. After adjustment for potential confounding, a 69% relative survival benefit was observed after receiving a therapeutic LP.

Raised intracranial pressure is common in cryptococcal meningitis, occurring in >60% of patients in sub-Saharan Africa [5, 15, 17]; thus, these findings may have a large impact on recovery from cryptococcal meningitis. Prior data suggest ICP can build up over time, and any rise may initially be asymptomatic [11, 15]. Therefore, reducing CSF volume before ICP has increased to symptomatic or detrimental levels is one possible explanation for improved survival after a therapeutic LP. In AIDS-related cryptococcosis, increased pressure primarily results from blockage of CSF drainage in the arachnoid villi and granulations by masses of cryptococcal cells or shed polysaccharide, inflammation, or a combination of these factors [11, 21–23]. Several studies detail a possible link between raised ICP and short-term mortality after cryptococcosis [11, 12, 15, 21]; however, no prior direct estimates exist of the effect of ICP management with therapeutic LPs on mortality. The evidence from this analysis provides a direct estimate of a survival benefit with therapeutic LPs and strongly supports the current treatment guidelines, which stress the importance of ICP management in cryptococcal meningitis.

The effect of therapeutic LPs did not appear to be relegated to only those with high baseline opening pressures, suggesting that all cryptococcal patients—regardless of initial opening pressure—may benefit from therapeutic LPs. However, as this subgroup analysis was small and likely underpowered, further studies of therapeutic LPs are needed to understand whether all patients would experience a survival benefit with therapeutic LPs during antifungal treatment.

Another important finding was that less CSF volume was removed during LPs in which the opening pressure was not measured. Additionally, patients in whom the opening pressure was not measured during the diagnostic LP were less likely to receive therapeutic LPs in follow-up. Among other differences that may exist for those without measured pressure, it is possible that the initial volume removed was insufficient to normalize pressure, partly explaining the increased mortality rate in this subgroup. Unfortunately, most patients throughout the world do not have the baseline CSF pressure measured because of limited awareness of the importance of measuring CSF opening pressure [24] or lack of access to manometers for accurate measurement [15, 25].

There is good evidence that intravenous tubing sets assembled to spinal needles coupled with a meter measuring stick are an accurate alternative to manometers to measure CSF opening pressure [17]; however, in the absence of a manometer or pressure measurement, the optimal timing and volume of CSF removed during repeat LPs is unclear. Recommendations are to initially remove 20 mL of CSF during the diagnostic LP and repeat therapeutic LPs daily, if needed based on symptoms [26]. One problem with this recommendation is that typically the *Cryptococcus* diagnosis is made after the initial diagnostic LP is complete, and the opportunity is missed to quickly remove greater volumes of CSF and reduce ICP. Our group in Uganda now routinely prescreens HIV-infected patients with subacute/chronic meningitis with the cryptococcal antigen lateral flow assay (Immy, Inc., Norman, Oklahoma) using fingerstick specimens or whole blood/plasma specimens at the bedside during the informed consent process prior to the lumbar puncture. For patients with a positive cryptococcal antigen, a manometer can be prioritized for use, and ICP can be normalized during the first LP a patient receives. Furthermore, an inability to control ICP may be unavoidable using a strategy based on reported symptoms, which, when compounded by the time demands of repeat LPs, make repeat therapeutic LPs inconsistent and infrequent, as our data suggest.

A limitation to our analysis is the potential for unmeasured confounding, as data on the daily clinical status of patients was unavailable for analysis, particularly prior to COAT trial randomization. Characteristics such as worsening headache or declines in mental status may have occurred more among individuals who died quickly. If these symptoms were also more common among those who did not receive an LP—perhaps because the patient or his/her attendant declined an LP, then uncontrolled confounding may be present. Although it is unknown whether such confounding exists, it is reasonable to assume that LPs were uniformly considered and recommended because the same clinician attended to patients within a site. This uniformity reduces the potential for confounding. One approach to assess for residual confounding was to evaluate the effect of therapeutic LPs stratified by baseline opening pressure, a strong indicator for undergoing additional LPs. The mortality trend was similar by baseline pressure strata providing some reassurance against residual confounding.

Further investigation of therapeutic LPs during cryptococcal meningitis therapy is warranted to see if the beneficial effect is uniformly experienced in different settings and to better understand the role of multiple therapeutic LPs. In our cohort therapeutic LPs were considered based on baseline CSF opening pressure and symptoms of raised ICP, whereas other studies have used a systematic approach when conducting scheduled therapeutic LPs for phase II trials to document the early fungicidal activity. These studies with scheduled LPs have failed to find associations between raised baseline ICP and increased mortality [4, 15]. Future trials should examine whether survival differs between a strategy to perform therapeutic LPs based on CSF opening pressures >250 mmH_2_O vs a strategy using a predefined schedule of lumbar punctures (eg, on days 1, 3, 7, and 14) for all patients.

In conclusion, our analysis supports the call for continued vigilance of ICP management during the window of high mortality shortly after diagnosis with HIV-associated cryptococcal meningitis, including improved access to manometers to monitor CSF pressures and the use of therapeutic LPs to reduce CSF pressure during antifungal therapy.

## Supplementary Data

Supplementary materials are available at *Clinical Infectious Diseases* online (http://cid.oxfordjournals.org). Supplementary materials consist of data provided by the author that are published to benefit the reader. The posted materials are not copyedited. The contents of all supplementary data are the sole responsibility of the authors. Questions or messages regarding errors should be addressed to the author.

Supplementary Data
