# Supplementary material for: The Effect of Therapeutic Lumbar Punctures on Acute Mortality From Cryptococcal Meningitis
Source: Clin Infect Dis. 2014 Jul 23;59(11):1607–14. doi: 10.1093/cid/ciu596 (PMC4441057; doi:10.1093/cid/ciu596)
Supplement: Supplementary Data [file supp_59_11_1607__index.html]

The Effect of Therapeutic Lumbar Punctures on Acute Mortality from Cryptococcal Meningitis — The Effect of Therapeutic Lumbar Punctures on Acute Mortality From Cryptococcal Meningitis — The Effect of Therapeutic Lumbar Punctures on Acute Mortality From Cryptococcal Meningitis — Supplementary Data 

# The Effect of Therapeutic Lumbar Punctures on Acute Mortality From Cryptococcal Meningitis

## Supplementary Data

Supplementary Data

**Files in this Data Supplement:**

- Supplementary Data - Docx file
