# Supplementary material for: The Effect of Therapeutic Lumbar Punctures on Acute Mortality From Cryptococcal Meningitis
Source: Clin Infect Dis. 2014 Jul 23;59(11):1607–14. doi: 10.1093/cid/ciu596 (PMC4441057; doi:10.1093/cid/ciu596)
Supplement: Supplementary Data [file supp_ciu596_ciu596supp.docx]

Appendix: Statistical analysis methods

Inverse Probability Weighting

To assess the effect of therapeutic lumbar punctures (LPs) on mortality from cryptococcal meningitis, a pooled Poisson model was run. A log link was used to estimate the relative risk of mortality among those who received at least one therapeutic LP compared to those who did not receive further LPs. Inverse probability weights were used to control for confounding. This method was chosen because of the potential for sparse data with the smaller sample size of the cohort.

Inverse probability weights were estimated from logistic regression models and weights were calculated for both the probability of exposure and the probability of being censored over the observation time. Stabilized weights for exposure and censoring were estimated and final stabilized weights (sw_i_), used in the model, were the product of the stabilized exposure and censoring weights (Robins, *Epidemiology*, 2000). Robust standard errors, using generalized estimating equations, were calculated to account for intra-subject correlation induced by the weighting scheme (Robins, *Epidemiology*, 2000). and to adjust for estimation of the relative risk from a logistic model (Zou, *Am J Epidemiology*, 2004).

Multiple Imputation for Missing Baseline Data

A Markov Chain Monte-Carlo process was used for multiple imputation and 40 full datasets were imputed. The imputation model included baseline demographics, CSF parameters, complete blood counts, symptoms of meningitis, vital signs, exposure to therapeutic LPs, and death. Exposure to therapeutic LPs and vital status were included in the imputation model to improve the prediction of missing parameters; however, no individuals were missing this information.
